# 5,7-Bis(1-benzothio­phen-2-yl)-2,3-dihydro­thieno[3,4-*b*][1,4]dioxine

**DOI:** 10.1107/S1600536808012324

**Published:** 2008-05-10

**Authors:** P. Sugumar, S. Ranjith, J. Arul Clement, A. K. Mohanakrishnan, M. N. Ponnuswamy

**Affiliations:** a Centre of Advanced Study in Crystallography and Biophysics, University of Madras, Guindy Campus, Chennai 600 025, India; b Department of Organic Chemistry, University of Madras, Guindy Campus, Chennai 600 025, India

## Abstract

In the title compound, C_22_H_14_O_2_S_3_, the dioxane ring is disordered over two sites [site occupancies = 0.623 (3) and 0.377 (3)]; both components adopt half-chair conformations. The two benzothio­phene ring systems are asymmetrically twisted away from the attached thio­phene ring [dihedral angles = 20.57 (3) and 6.70 (3)°] and are oriented at an angle of 26.83 (3)°. No significant hydrogen bonding or π–π inter­actions are observed in the crystal structure.

## Related literature

For related literature, see: Cohen *et al.* (1977 ▶); Csaszar & Morvay (1983 ▶); Dzhurayev *et al.* (1992 ▶); EI-Maghraby *et al.* (1984 ▶); Gewald *et al.* (1996 ▶); Lakshmi *et al.* (1985 ▶); Pellis & West (1968 ▶). For the synthesis, see: Amaladass *et al.* (2007 ▶).
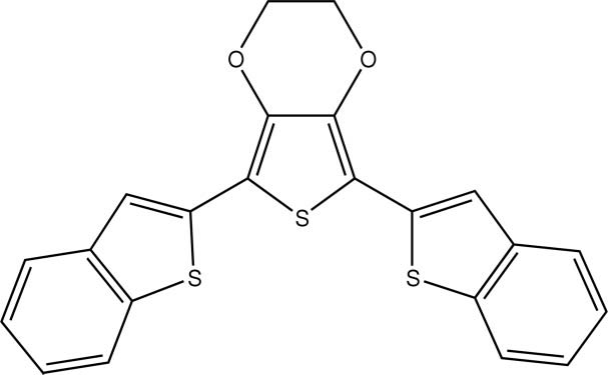


## Experimental

### 

#### Crystal data

C_22_H_14_O_2_S_3_
*M* *_r_* = 406.51
Monoclinic, 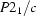

*a* = 16.1602 (5) Å
*b* = 8.3524 (3) Å
*c* = 14.1814 (4) Å
β = 107.428 (2)°
*V* = 1826.28 (10) Å^3^
*Z* = 4
Mo *K*α radiation
μ = 0.42 mm^−1^
*T* = 293 (2) K
0.15 × 0.13 × 0.10 mm

#### Data collection

Bruker Kappa APEXII area-detector diffractometer
Absorption correction: none
26571 measured reflections
7059 independent reflections
4551 reflections with *I* > 2σ(*I*)
*R*_int_ = 0.028

#### Refinement

*R*[*F* ^2^ > 2σ(*F* ^2^)] = 0.044
*wR*(*F* ^2^) = 0.142
*S* = 0.99
7059 reflections
251 parameters
3 restraints
H-atom parameters constrained
Δρ_max_ = 0.61 e Å^−3^
Δρ_min_ = −0.43 e Å^−3^

### 

Data collection: *APEX2* (Bruker, 2004 ▶); cell refinement: *APEX2*; data reduction: *SAINT* (Bruker, 2004 ▶); program(s) used to solve structure: *SHELXS97* (Sheldrick, 2008 ▶); program(s) used to refine structure: *SHELXL97* (Sheldrick, 2008 ▶); molecular graphics: *PLATON* (Spek, 2003 ▶); software used to prepare material for publication: *SHELXL97* and *PARST* (Nardelli, 1995 ▶).

## Supplementary Material

Crystal structure: contains datablocks I, global. DOI: 10.1107/S1600536808012324/ci2577sup1.cif

Structure factors: contains datablocks I. DOI: 10.1107/S1600536808012324/ci2577Isup2.hkl

Additional supplementary materials:  crystallographic information; 3D view; checkCIF report

## Figures and Tables

**Fig. 1.**
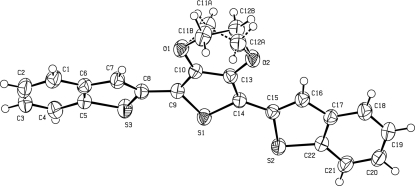
The molecular structure of the title compound, showing 30% probability displacement ellipsoids. Both disorder components are shown.

## supplementary crystallographic information

### Comment

Sulfur containing Schiff bases (Pellis & West, 1968; Cohen *et al.*, 1977; Csaszar & Morvay,1983; Lakshmi *et al.*, 1985) and their thiophen derivatives (EI-Maghraby *et al.*, 1984; Dzhurayev *et al.*, 1992) possess pharmacological activities such as anti-bacterial, anti-cancer, anti-inflammatory and anti-toxic properties (Gewald *et al.*, 1996). Benzo[*b*]thiophene analogs have been shown to possess interesting estrogenic and antiestrogenic effects. We report here the crystal structure of the title compound.

The C1—C8/S3 and C15—C22/S2 benzothiophene ring systems are essentially planar and are oriented at angles of 20.57 (3)° and 6.70 (3)°, respectively, with respect to the thiophene ring. The dihedral angle between the two benzothiophene ring systems is 26.83 (3)°. Both the major and minor conformers of the disordered dioxane ring adopt half-chair conformations. The crystal packing is stabilized by van der Waals forces.

### Experimental

The title compound was prepared according to the procedure reported by Amaladass *et al.* (2007). Single crystals suitable for X-ray analysis were obtained by slow evaporation method.

### Refinement

The methylene C atoms of the dioxane ring are disordered over two positions (C11A/C11B and C12A/C12B) with refined occupancies of 0.623 (3) and 0.377 (3). The corresponding bond distances involving the disordered atoms were restrained to be equal, and also the same U^ij^ parameters were used for atoms C11A and C11B, and C12A and C12B. H atoms were positioned geometrically (C—H = 0.93 Å or 0.97 Å) and were treated as riding on their parent atoms, with *U*_iso_(H) = 1.2*U*_eq_(C).

### Crystal data

**Table:**

C_22_H_14_O_2_S_3_	*F*_000_ = 840
*M**_r_* = 406.51	*D*_x_ = 1.478 Mg m^−^^3^
Monoclinic, *P*2_1_/*c*	Mo *K*α radiation λ = 0.71073 Å
Hall symbol: -P 2ybc	Cell parameters from 4583 reflections
*a* = 16.1602 (5) Å	θ = 2.8–33.8º
*b* = 8.3524 (3) Å	µ = 0.42 mm^−^^1^
*c* = 14.1814 (4) Å	*T* = 293 (2) K
β = 107.428 (2)º	Block, light green
*V* = 1826.28 (10) Å^3^	0.15 × 0.13 × 0.10 mm
*Z* = 4	

### Data collection

**Table:**

Bruker Kappa APEXII area-detector diffractometer	4551 reflections with *I* > 2σ(*I*)
Radiation source: fine-focus sealed tube	*R*_int_ = 0.028
Monochromator: graphite	θ_max_ = 33.4º
*T* = 293(2) K	θ_min_ = 2.8º
ω and φ scans	*h* = −24→24
Absorption correction: none	*k* = −12→10
26571 measured reflections	*l* = −21→19
7059 independent reflections	

### Refinement

**Table:**

Refinement on *F*^2^	Secondary atom site location: difference Fourier map
Least-squares matrix: full	Hydrogen site location: inferred from neighbouring sites
*R*[*F*^2^ > 2σ(*F*^2^)] = 0.044	H-atom parameters constrained
*wR*(*F*^2^) = 0.143	*w* = 1/[σ^2^(*F*_o_^2^) + (0.0749*P*)^2^ + 0.444*P*] where *P* = (*F*_o_^2^ + 2*F*_c_^2^)/3
*S* = 0.99	(Δ/σ)_max_ < 0.001
7059 reflections	Δρ_max_ = 0.61 e Å^−^^3^
251 parameters	Δρ_min_ = −0.43 e Å^−^^3^
3 restraints	Extinction correction: none
Primary atom site location: structure-invariant direct methods	

### Special details

**Table:**

Geometry. All e.s.d.'s (except the e.s.d. in the dihedral angle between two l.s. planes) are estimated using the full covariance matrix. The cell e.s.d.'s are taken into account individually in the estimation of e.s.d.'s in distances, angles and torsion angles; correlations between e.s.d.'s in cell parameters are only used when they are defined by crystal symmetry. An approximate (isotropic) treatment of cell e.s.d.'s is used for estimating e.s.d.'s involving l.s. planes.
Refinement. Refinement of *F*^2^ against ALL reflections. The weighted *R*-factor *wR* and goodness of fit *S* are based on *F*^2^, conventional *R*-factors *R* are based on *F*, with *F* set to zero for negative *F*^2^. The threshold expression of *F*^2^ > σ(*F*^2^) is used only for calculating *R*-factors(gt) *etc*. and is not relevant to the choice of reflections for refinement. *R*-factors based on *F*^2^ are statistically about twice as large as those based on *F*, and *R*- factors based on ALL data will be even larger.

### Fractional atomic coordinates and isotropic or equivalent isotropic displacement parameters (Å^2^)

**Table:**

	*x*	*y*	*z*	*U*_iso_*/*U*_eq_	Occ. (<1)
C1	−0.28863 (11)	0.4031 (2)	−0.01894 (14)	0.0499 (4)	
H1	−0.2949	0.3569	−0.0804	0.060*	
C2	−0.35994 (12)	0.4572 (3)	0.00498 (16)	0.0578 (5)	
H2	−0.4145	0.4490	−0.0412	0.069*	
C3	−0.35223 (12)	0.5241 (2)	0.09694 (18)	0.0597 (5)	
H3	−0.4017	0.5590	0.1115	0.072*	
C4	−0.27258 (12)	0.5395 (2)	0.16681 (16)	0.0533 (4)	
H4	−0.2674	0.5837	0.2285	0.064*	
C5	−0.19971 (10)	0.48665 (19)	0.14227 (13)	0.0419 (3)	
C6	−0.20599 (10)	0.41809 (19)	0.05007 (12)	0.0400 (3)	
C7	−0.12301 (10)	0.3765 (2)	0.03966 (13)	0.0430 (3)	
H7	−0.1153	0.3287	−0.0164	0.052*	
C8	−0.05619 (10)	0.41564 (19)	0.12239 (11)	0.0374 (3)	
C9	0.03586 (9)	0.39615 (18)	0.13747 (11)	0.0364 (3)	
C10	0.07580 (9)	0.29651 (18)	0.08760 (11)	0.0357 (3)	
C11A	0.08383 (17)	0.1322 (5)	−0.0432 (2)	0.0502 (8)	0.623 (3)
H11A	0.0937	0.2125	−0.0881	0.060*	0.623 (3)
H11B	0.0543	0.0420	−0.0820	0.060*	0.623 (3)
C12A	0.16828 (17)	0.0790 (3)	0.0260 (2)	0.0485 (6)	0.623 (3)
H12A	0.1574	−0.0005	0.0707	0.058*	0.623 (3)
H12B	0.2022	0.0283	−0.0117	0.058*	0.623 (3)
C11B	0.0915 (3)	0.0850 (5)	−0.0061 (4)	0.0502 (8)	0.377 (3)
H11C	0.0623	0.0295	−0.0670	0.060*	0.377 (3)
H11D	0.1046	0.0059	0.0464	0.060*	0.377 (3)
C12B	0.1754 (3)	0.1472 (7)	−0.0147 (2)	0.0485 (6)	0.377 (3)
H12C	0.2092	0.0622	−0.0318	0.058*	0.377 (3)
H12D	0.1658	0.2309	−0.0643	0.058*	0.377 (3)
C13	0.16712 (9)	0.30254 (18)	0.12251 (11)	0.0365 (3)	
C14	0.19858 (9)	0.40987 (19)	0.19816 (11)	0.0362 (3)	
C15	0.28812 (9)	0.44752 (19)	0.24924 (11)	0.0352 (3)	
C16	0.36041 (10)	0.3755 (2)	0.23942 (12)	0.0416 (3)	
H16	0.3587	0.2893	0.1973	0.050*	
C17	0.43920 (10)	0.44442 (19)	0.29966 (11)	0.0380 (3)	
C18	0.52506 (11)	0.4008 (2)	0.30912 (15)	0.0512 (4)	
H18	0.5366	0.3159	0.2725	0.061*	
C19	0.59199 (11)	0.4840 (2)	0.37253 (15)	0.0521 (4)	
H19	0.6489	0.4558	0.3781	0.062*	
C20	0.57576 (11)	0.6105 (2)	0.42876 (14)	0.0483 (4)	
H20	0.6220	0.6645	0.4722	0.058*	
C21	0.49236 (11)	0.6563 (2)	0.42076 (13)	0.0469 (4)	
H21	0.4816	0.7414	0.4578	0.056*	
C22	0.42398 (9)	0.57260 (19)	0.35592 (11)	0.0374 (3)	
O1	0.03162 (7)	0.19777 (15)	0.01304 (9)	0.0476 (3)	
O2	0.21883 (7)	0.21067 (15)	0.08407 (9)	0.0467 (3)	
S1	0.11311 (2)	0.50367 (5)	0.22588 (3)	0.04068 (11)	
S2	0.31369 (3)	0.60648 (5)	0.33305 (3)	0.04677 (12)	
S3	−0.09260 (3)	0.49989 (6)	0.21576 (3)	0.04834 (13)	

### Atomic displacement parameters (Å^2^)

**Table:**

	*U*^11^	*U*^22^	*U*^33^	*U*^12^	*U*^13^	*U*^23^
C1	0.0415 (8)	0.0560 (10)	0.0467 (9)	−0.0071 (7)	0.0047 (7)	0.0089 (8)
C2	0.0338 (8)	0.0654 (12)	0.0667 (12)	−0.0030 (8)	0.0037 (8)	0.0162 (10)
C3	0.0355 (8)	0.0585 (11)	0.0856 (15)	0.0057 (8)	0.0188 (9)	0.0054 (10)
C4	0.0418 (9)	0.0515 (10)	0.0684 (12)	0.0037 (7)	0.0192 (8)	−0.0063 (9)
C5	0.0336 (7)	0.0393 (8)	0.0529 (9)	−0.0002 (6)	0.0131 (6)	0.0001 (7)
C6	0.0355 (7)	0.0390 (8)	0.0445 (8)	−0.0019 (6)	0.0103 (6)	0.0061 (6)
C7	0.0352 (7)	0.0473 (9)	0.0470 (8)	0.0003 (6)	0.0132 (6)	0.0079 (7)
C8	0.0331 (7)	0.0393 (8)	0.0401 (7)	0.0004 (5)	0.0114 (5)	0.0008 (6)
C9	0.0316 (6)	0.0395 (8)	0.0378 (7)	−0.0003 (5)	0.0098 (5)	0.0003 (6)
C10	0.0332 (6)	0.0376 (7)	0.0353 (7)	−0.0025 (5)	0.0086 (5)	−0.0015 (6)
C11A	0.0450 (11)	0.070 (2)	0.0369 (17)	−0.0104 (12)	0.0148 (13)	−0.0198 (14)
C12A	0.0432 (11)	0.0540 (18)	0.0478 (15)	0.0009 (11)	0.0128 (11)	−0.0150 (11)
C11B	0.0450 (11)	0.070 (2)	0.0369 (17)	−0.0104 (12)	0.0148 (13)	−0.0198 (14)
C12B	0.0432 (11)	0.0540 (18)	0.0478 (15)	0.0009 (11)	0.0128 (11)	−0.0150 (11)
C13	0.0340 (7)	0.0378 (8)	0.0383 (7)	0.0019 (5)	0.0116 (5)	−0.0024 (6)
C14	0.0317 (6)	0.0395 (8)	0.0368 (7)	−0.0001 (5)	0.0095 (5)	−0.0018 (6)
C15	0.0338 (7)	0.0372 (7)	0.0349 (7)	−0.0016 (5)	0.0106 (5)	−0.0026 (6)
C16	0.0363 (7)	0.0423 (8)	0.0461 (8)	−0.0022 (6)	0.0122 (6)	−0.0089 (7)
C17	0.0338 (7)	0.0402 (8)	0.0411 (7)	−0.0023 (6)	0.0130 (6)	−0.0008 (6)
C18	0.0364 (8)	0.0547 (10)	0.0645 (11)	0.0013 (7)	0.0181 (7)	−0.0109 (8)
C19	0.0328 (8)	0.0577 (11)	0.0659 (11)	−0.0027 (7)	0.0153 (7)	−0.0001 (9)
C20	0.0360 (8)	0.0509 (10)	0.0535 (9)	−0.0103 (7)	0.0066 (7)	0.0011 (8)
C21	0.0402 (8)	0.0486 (9)	0.0495 (9)	−0.0081 (7)	0.0096 (7)	−0.0090 (7)
C22	0.0324 (7)	0.0401 (8)	0.0394 (7)	−0.0029 (6)	0.0103 (5)	−0.0005 (6)
O1	0.0379 (6)	0.0534 (7)	0.0495 (6)	−0.0055 (5)	0.0100 (5)	−0.0169 (5)
O2	0.0355 (5)	0.0520 (7)	0.0520 (6)	0.0028 (5)	0.0121 (5)	−0.0160 (5)
S1	0.03413 (18)	0.0475 (2)	0.0409 (2)	−0.00031 (15)	0.01199 (14)	−0.00916 (16)
S2	0.03469 (19)	0.0501 (2)	0.0539 (2)	−0.00041 (16)	0.01081 (16)	−0.01687 (18)
S3	0.0361 (2)	0.0621 (3)	0.0464 (2)	0.00043 (17)	0.01175 (16)	−0.01084 (19)

### Geometric parameters (Å, °)

**Table:**

C1—C2	1.372 (3)	C11B—O1	1.434 (2)
C1—C6	1.405 (2)	C11B—C12B	1.490 (3)
C1—H1	0.93	C11B—H11C	0.97
C2—C3	1.390 (3)	C11B—H11D	0.97
C2—H2	0.93	C12B—O2	1.465 (3)
C3—C4	1.375 (3)	C12B—H12C	0.97
C3—H3	0.93	C12B—H12D	0.97
C4—C5	1.396 (2)	C13—O2	1.3627 (18)
C4—H4	0.93	C13—C14	1.373 (2)
C5—C6	1.403 (2)	C14—C15	1.445 (2)
C5—S3	1.7361 (17)	C14—S1	1.7325 (15)
C6—C7	1.435 (2)	C15—C16	1.358 (2)
C7—C8	1.375 (2)	C15—S2	1.7470 (15)
C7—H7	0.93	C16—C17	1.425 (2)
C8—C9	1.447 (2)	C16—H16	0.93
C8—S3	1.7496 (16)	C17—C22	1.400 (2)
C9—C10	1.372 (2)	C17—C18	1.401 (2)
C9—S1	1.7318 (15)	C18—C19	1.371 (3)
C10—O1	1.3622 (18)	C18—H18	0.93
C10—C13	1.410 (2)	C19—C20	1.395 (3)
C11A—O1	1.433 (2)	C19—H19	0.93
C11A—C12A	1.490 (3)	C20—C21	1.373 (2)
C11A—H11A	0.97	C20—H20	0.93
C11A—H11B	0.97	C21—C22	1.395 (2)
C12A—O2	1.467 (2)	C21—H21	0.93
C12A—H12A	0.97	C22—S2	1.7365 (15)
C12A—H12B	0.97		
			
C2—C1—C6	119.47 (18)	C12B—C11B—H11D	107.8
C2—C1—H1	120.3	H11C—C11B—H11D	107.2
C6—C1—H1	120.3	O2—C12B—C11B	103.6 (3)
C1—C2—C3	121.26 (17)	O2—C12B—H12C	111.0
C1—C2—H2	119.4	C11B—C12B—H12C	111.0
C3—C2—H2	119.4	O2—C12B—H12D	111.0
C4—C3—C2	121.00 (18)	C11B—C12B—H12D	111.0
C4—C3—H3	119.5	H12C—C12B—H12D	109.0
C2—C3—H3	119.5	O2—C13—C14	123.51 (13)
C3—C4—C5	117.93 (19)	O2—C13—C10	122.83 (13)
C3—C4—H4	121.0	C14—C13—C10	113.66 (13)
C5—C4—H4	121.0	C13—C14—C15	127.90 (14)
C4—C5—C6	122.04 (16)	C13—C14—S1	109.79 (11)
C4—C5—S3	126.46 (15)	C15—C14—S1	122.30 (12)
C6—C5—S3	111.48 (12)	C16—C15—C14	127.96 (14)
C5—C6—C1	118.29 (16)	C16—C15—S2	111.78 (11)
C5—C6—C7	112.69 (14)	C14—C15—S2	120.24 (11)
C1—C6—C7	129.00 (17)	C15—C16—C17	113.66 (14)
C8—C7—C6	111.96 (15)	C15—C16—H16	123.2
C8—C7—H7	124.0	C17—C16—H16	123.2
C6—C7—H7	124.0	C22—C17—C18	118.81 (15)
C7—C8—C9	127.53 (14)	C22—C17—C16	111.87 (13)
C7—C8—S3	112.65 (12)	C18—C17—C16	129.32 (16)
C9—C8—S3	119.82 (11)	C19—C18—C17	119.69 (17)
C10—C9—C8	127.84 (14)	C19—C18—H18	120.2
C10—C9—S1	109.84 (11)	C17—C18—H18	120.2
C8—C9—S1	122.33 (12)	C18—C19—C20	120.82 (16)
O1—C10—C9	123.31 (13)	C18—C19—H19	119.6
O1—C10—C13	122.97 (13)	C20—C19—H19	119.6
C9—C10—C13	113.69 (13)	C21—C20—C19	120.79 (16)
O1—C11A—C12A	108.9 (2)	C21—C20—H20	119.6
O1—C11A—H11A	109.9	C19—C20—H20	119.6
C12A—C11A—H11A	109.9	C20—C21—C22	118.62 (17)
O1—C11A—H11B	109.9	C20—C21—H21	120.7
C12A—C11A—H11B	109.9	C22—C21—H21	120.7
H11A—C11A—H11B	108.3	C21—C22—C17	121.27 (15)
O2—C12A—C11A	113.1 (2)	C21—C22—S2	127.40 (13)
O2—C12A—H12A	109.0	C17—C22—S2	111.34 (11)
C11A—C12A—H12A	109.0	C10—O1—C11A	113.88 (16)
O2—C12A—H12B	109.0	C10—O1—C11B	108.6 (3)
C11A—C12A—H12B	109.0	C13—O2—C12B	114.2 (2)
H12A—C12A—H12B	107.8	C13—O2—C12A	109.99 (15)
O1—C11B—C12B	117.9 (4)	C9—S1—C14	92.98 (7)
O1—C11B—H11C	107.8	C22—S2—C15	91.35 (7)
C12B—C11B—H11C	107.8	C5—S3—C8	91.20 (8)
O1—C11B—H11D	107.8		
			
C6—C1—C2—C3	−1.2 (3)	C15—C16—C17—C18	179.46 (17)
C1—C2—C3—C4	0.6 (3)	C22—C17—C18—C19	−0.2 (3)
C2—C3—C4—C5	0.4 (3)	C16—C17—C18—C19	−179.69 (18)
C3—C4—C5—C6	−0.7 (3)	C17—C18—C19—C20	0.8 (3)
C3—C4—C5—S3	177.48 (15)	C18—C19—C20—C21	−1.0 (3)
C4—C5—C6—C1	0.1 (2)	C19—C20—C21—C22	0.7 (3)
S3—C5—C6—C1	−178.33 (13)	C20—C21—C22—C17	−0.1 (3)
C4—C5—C6—C7	178.37 (16)	C20—C21—C22—S2	179.76 (14)
S3—C5—C6—C7	−0.02 (18)	C18—C17—C22—C21	−0.2 (2)
C2—C1—C6—C5	0.8 (2)	C16—C17—C22—C21	179.43 (15)
C2—C1—C6—C7	−177.15 (17)	C18—C17—C22—S2	179.96 (13)
C5—C6—C7—C8	−0.8 (2)	C16—C17—C22—S2	−0.43 (18)
C1—C6—C7—C8	177.27 (16)	C9—C10—O1—C11A	166.6 (2)
C6—C7—C8—C9	−177.96 (15)	C13—C10—O1—C11A	−15.2 (3)
C6—C7—C8—S3	1.29 (18)	C9—C10—O1—C11B	−166.3 (3)
C7—C8—C9—C10	−20.4 (3)	C13—C10—O1—C11B	11.9 (3)
S3—C8—C9—C10	160.45 (14)	C12A—C11A—O1—C10	43.7 (3)
C7—C8—C9—S1	159.31 (14)	C12A—C11A—O1—C11B	−39.6 (5)
S3—C8—C9—S1	−19.89 (18)	C12B—C11B—O1—C10	−46.7 (5)
C8—C9—C10—O1	0.2 (3)	C12B—C11B—O1—C11A	60.0 (5)
S1—C9—C10—O1	−179.48 (12)	C14—C13—O2—C12B	−160.2 (3)
C8—C9—C10—C13	−178.15 (15)	C10—C13—O2—C12B	19.4 (3)
S1—C9—C10—C13	2.15 (17)	C14—C13—O2—C12A	163.91 (19)
O1—C11A—C12A—O2	−62.3 (4)	C10—C13—O2—C12A	−16.4 (2)
O1—C11B—C12B—O2	64.4 (6)	C11B—C12B—O2—C13	−46.1 (4)
O1—C10—C13—O2	0.4 (2)	C11B—C12B—O2—C12A	43.8 (2)
C9—C10—C13—O2	178.81 (14)	C11A—C12A—O2—C13	47.1 (3)
O1—C10—C13—C14	−179.88 (14)	C11A—C12A—O2—C12B	−56.9 (4)
C9—C10—C13—C14	−1.5 (2)	C10—C9—S1—C14	−1.77 (12)
O2—C13—C14—C15	0.8 (3)	C8—C9—S1—C14	178.51 (13)
C10—C13—C14—C15	−178.84 (15)	C13—C14—S1—C9	0.95 (13)
O2—C13—C14—S1	179.79 (12)	C15—C14—S1—C9	179.96 (13)
C10—C13—C14—S1	0.11 (17)	C21—C22—S2—C15	−179.22 (16)
C13—C14—C15—C16	−6.2 (3)	C17—C22—S2—C15	0.63 (13)
S1—C14—C15—C16	174.99 (14)	C16—C15—S2—C22	−0.69 (13)
C13—C14—C15—S2	171.90 (13)	C14—C15—S2—C22	−179.06 (13)
S1—C14—C15—S2	−6.92 (19)	C4—C5—S3—C8	−177.68 (17)
C14—C15—C16—C17	178.79 (15)	C6—C5—S3—C8	0.63 (13)
S2—C15—C16—C17	0.57 (19)	C7—C8—S3—C5	−1.11 (13)
C15—C16—C17—C22	−0.1 (2)	C9—C8—S3—C5	178.20 (13)
